# Jejunoileal Atresia: A National Cohort Study

**DOI:** 10.3389/fped.2021.665022

**Published:** 2021-05-31

**Authors:** Andrea Schmedding, Martin Hutter, Stefan Gfroerer, Udo Rolle

**Affiliations:** ^1^Department of Pediatric Surgery and Pediatric Urology, University Hospital, Goethe University Frankfurt, Frankfurt am Main, Germany; ^2^Department of Pediatric Surgery, Helios-Clinic Berlin-Buch, Berlin, Germany

**Keywords:** jejunoileal atresia, stoma, outcome, small bowel, short bowel, children

## Abstract

**Purpose:** Jejunoileal atresia (JIA) is a rare disease. We aimed to determine the overall incidence of this malformation and associated malformations in a national cohort. Furthermore, we compared the treatment results of this cohort with the current literature.

**Methods:** Data from the major health insurance company, which covers ~30% of the German population, were analyzed. All patients with ICD-10-Code Q41.1-9 (atresia of jejunum, ileum, other parts and not designated parts of the small bowel) who underwent any surgical procedure for small bowel were analyzed in a 10-year period between 2007 and 2016.

**Results:** A total of 435 patients were included in the study. The incidence was 2.1 per 10,000 live births. The male:female ratio was 1:2. Sixty-four percent were premature, 21% had associated cardiac anomalies, 16% had abdominal wall defects, 7% had urogenital malformations, and 7% had cystic fibrosis. Sixty percent of all patients with jejunoileal atresia, 57% of patients with accompanying abdominal wall defects and 72% of patients with associated cystic fibrosis required ostomy as the initial procedure. In 25% of all patients, only one intestinal operation was coded. In 39% of patients, two operations were coded. Twelve percent of all patients required feeding gastrostomy or jejunostomy. Sixteen percent of all patients presented with liver-related complications, i.e., cholestasis or liver insufficiency. Six patients underwent an intestinal lengthening procedure (2 Bianchi, 4 STEP). In five patients, initial lengthening was performed within 1 year after the first intestinal operation. Mortality until 1 year after initial surgery was 5%. Of those who died, 88% were premature, 34% had cardiac anomalies and 16% had abdominal wall defects. None had cystic fibrosis. Patients with ostomy significantly more often needed operative central venous line or operative feeding tube. Short bowel was coded significantly more often in these patients.

**Conclusion:** Patients with JIA present with low mortality. The rate of ostomies is higher than in literature. To give clinical recommendations for the initial surgical approach, further clinical research is needed.

## Introduction

Jejunoileal atresia is a rare disease. The prevalence was reported to be between 0.3 and 1.1 per 10,000 births (1) in Europe. Many children are born premature (2). Frequent concomitant diseases include cardiac anomalies, defects of the abdominal wall and cystic fibrosis. Initial surgery was performed as primary anastomosis or ostomy. Depending on the type of atresia and associated anomalies, the respective children might do well, whereas others suffer from relevant problems, such as feeding issues, short bowel syndrome or liver insufficiency (3).

There is currently no registry for patients with small bowel atresia in Germany, and a large patient group has not been reported.

We have successfully employed health insurance data for epidemiological studies (4). Therefore, the aim of this study was to investigate the epidemiology of JIA in a national cohort using health insurance data.

In Germany, people can choose between different health insurance companies. The database of the major health insurance company in Germany (AOK) was retrospectively analyzed in our study. The AOK covers ~30% of the German population.

Their database contains personal data and data on inpatient and outpatient medical treatments, which the insurance has to reimburse. Data on medical treatments include codes for diagnoses based on the International Statistical Classification of Diseases and Related Health Problems–German modification (ICD-10-GM) (5) and codes for procedures based on the German procedure classification (OPS-codes) (6) together with the date of surgery as well as dates of admission and discharge.

We aimed to further assess concomitant diseases and outcome parameters of this national cohort to compare the results with the available data from the literature.

## Materials and Methods

In Germany in 2011, 87% of the inhabitants were insured under statutory health insurance (SHI) (7, 8). The AOK is the largest statutory health insurance company in the country; it has branches all over the country and covers approximately one-third of German patients. The rate of insured people <15 years of age are the same in AOK and all health insured people (9). An analyse between children insured at different companies showed no difference regarding sex and mean age. Children insured at AOK had more often two and more siblings and had more often migrant background. The study also indicated a lower socioeconomic status of insured people at AOK compared to other public and private health insurance companies (10). This must be taken into consideration when analyzing the data obtained from AOK.

All patients who were born between 2007 and 2016 and who were clients of AOK with a diagnosis of atresia of the small bowel were identified by the ICD-10 codes Q41.1 (atresia of jejunum), Q41.2 (atresia of ileum), Q41.8 (atresia of other specified parts of the small bowel), and Q41.9 (atresia of not designated parts of the small bowel). While the codes Q41.1, 2 and 9 are clearly related to anatomic or clinical situations the use or Q41.8 is not further specified in the German coding catalog (5). Those who had any surgical procedure of the small bowel, except the duodenum, coded by OPS-Codes 5-45, 5-46, 5-47, 5-48, and 5-49 were analyzed further. For each patient, the following parameters were obtained: year and months of birth, sex, and all inpatient stays while insured with AOK together with the medical data (diagnosis, procedures, and day of surgery). It is important to mention that several patients had multiple diagnoses.

Associated malformations of the gastrointestinal tract were included if there was an associated surgical procedure coded. Other associated anomalies were included as they were coded.

For the calculation of the incidence numbers of births in Germany were obtained from the Federal Statistical Bureau of Germany for the years 2007 to 2016 (11). There were 6,973,390 children born during these 10 years.

In Germany, the utilization of health insurance data for scientific research is regulated by the Code of Social Law. Data access was legally approved by the Scientific Institute of the AOK (WidO). The study uses only anonymized administrative claims data that never involved patients directly. The analysis of secondary data did not need Institutional Review Board approval as stated in the guidelines for Good Practice of Secondary Data Analysis (GPS) (12) and was confirmed by the local ethic committee.

### Statistical Analysis

Data were analyzed with descriptive statistics as the frequencies and percentages or the medians with quartiles and ranges for categorical and continuous variables, respectively.

Fisher's exact test was used for the analysis of the associated anomalies. The distribution of follow-up was calculated using the reverse Kaplan-Meier estimate. Mortality was analyzed using the Kaplan-Meier estimate. Survival time was counted from the day of initial surgery. We used the non-parametric cumulative incidence function of Fine and Gray (13) for length of hospital stay (LOS) analysis. In-house mortality was regarded as a competing risk for discharge (14).

Analyses were performed using R statistical software version 4.0.2 (15).

## Results

We identified 571 patients born between 2007 and 2016 with a diagnosis of congenital atresia or stenosis of the small bowel, of whom 435 underwent a corresponding procedure for the small bowel. Only patients with the diagnosis of small bowel atresia and corresponding procedure were taken for the analysis. There were 42 patients with necrotizing enterocolitis (NEC) as secondary diagnosis. In these patients the procedures for small bowel could not be related to a specific diagnosis so they were analyzed separately.

There were 235 boys and 200 girls altogether, and 209 boys and 184 girls for the group without NEC. The male:female ratio is 1.18:1 for all patients and 1.14:1 for the group without NEC. We calculated the incidence as 2.1 per 10,000 livebirths (1:4,809) for all patients and 1.9 per 10,000 livebirths (1:5,323) for the group without NEC.

**Figure 1:**
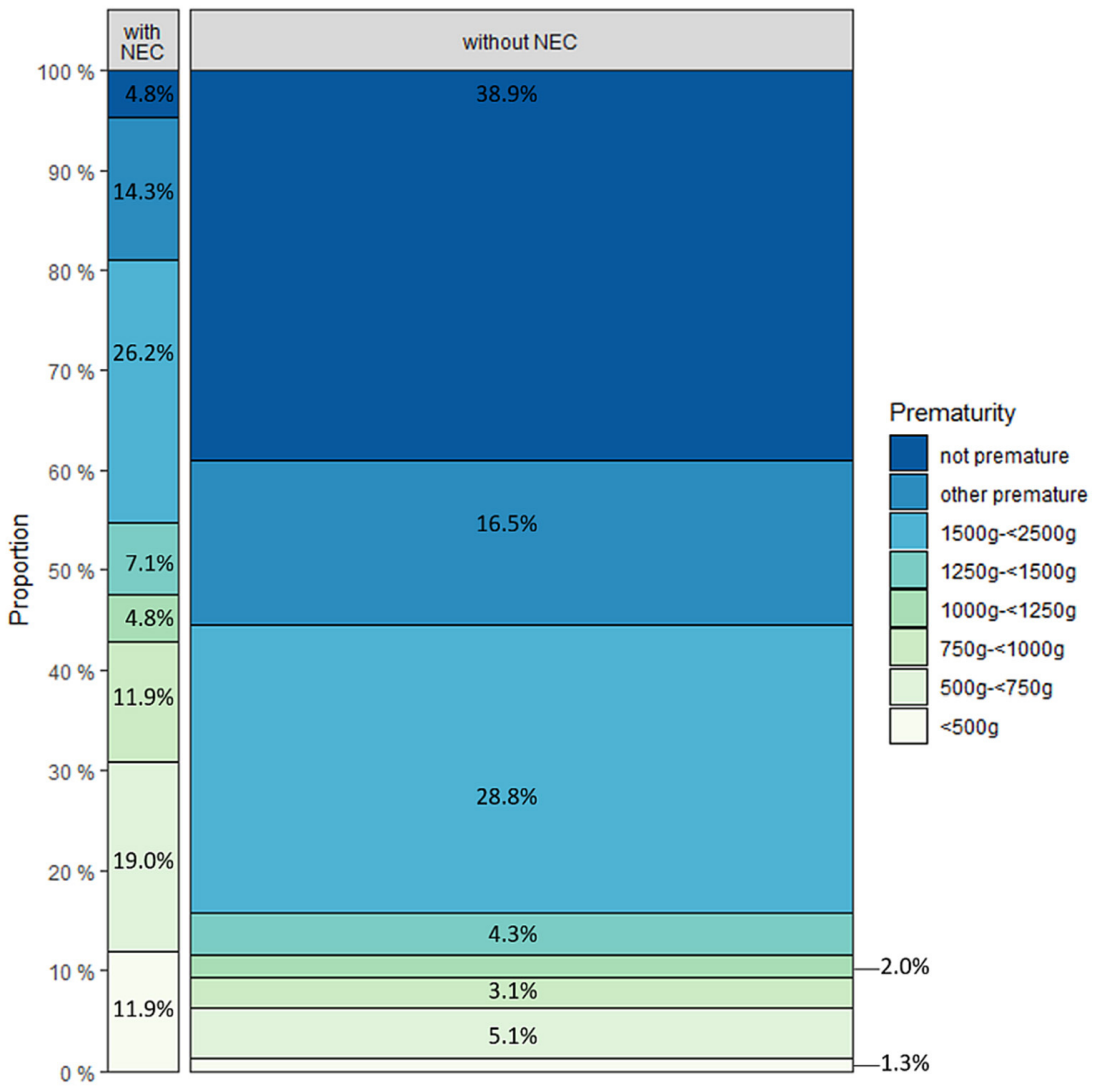
Distribution of patients with (*n* = 42) and without (*n* = 393) necrotizing enterocolitis regarding pre-maturity.

**Figure 2:**
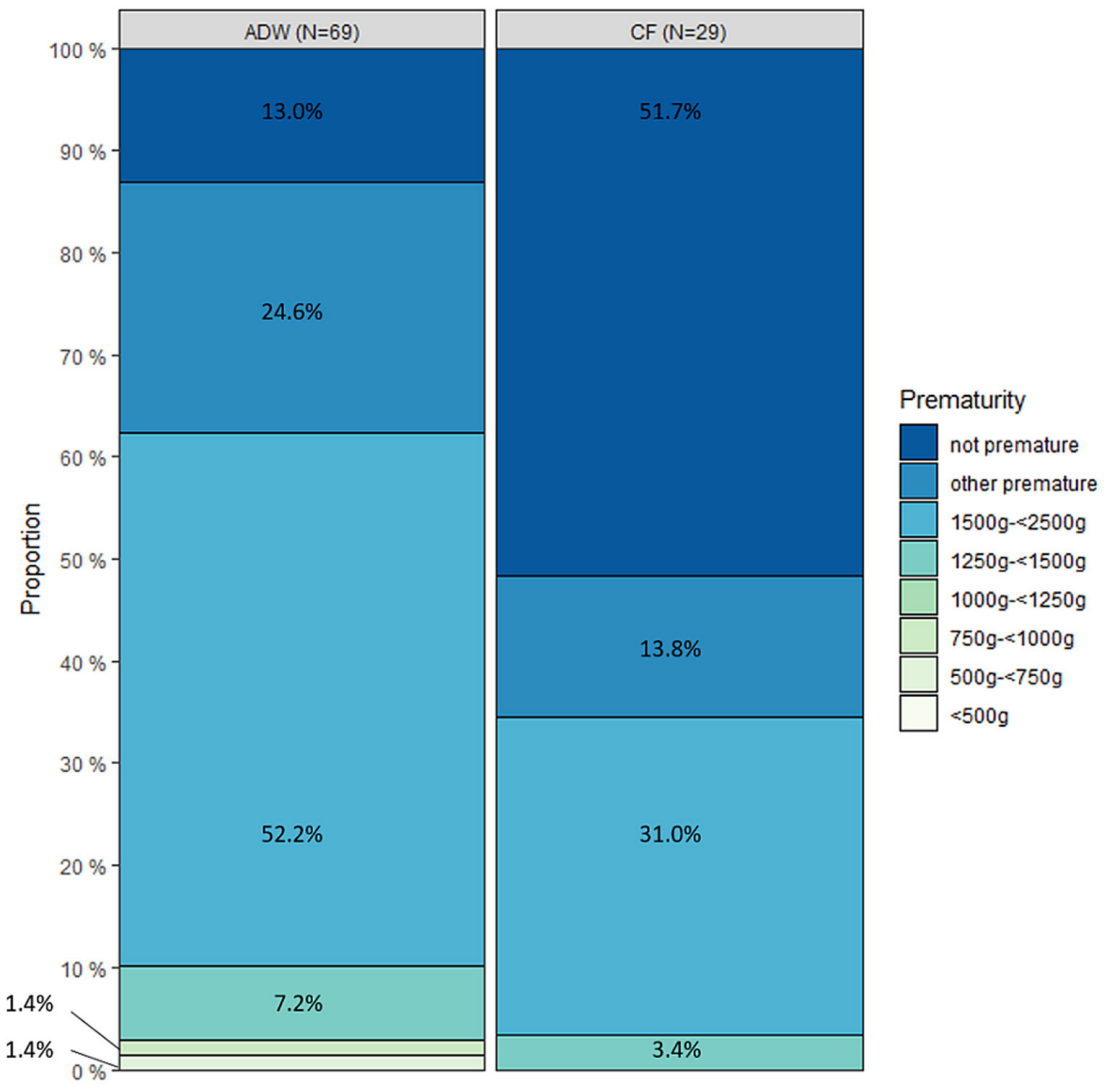
Distribution of patients with abdominal wall defect (AWD) (*n* = 69) and cystic fibrosis (CF) (*n* = 29) regarding pre-maturity.

Associated anomalies are listed in Table 1. The main associated diagnoses were prematurity (64.4%), cardiac malformations (21.1%) and defects of the abdominal wall (15.9%). Cystic fibrosis was present in 7% of the patients. The further analysis of prematurity in the different subgroups is provided in Figures 1, 2.

**Table 1:** Associated anomalies, pre-maturity of the children with atresia of small bowel together with outcome parameters.

	**Patients**	**Mortality 1-y after initial surgery**		**LOS**		**Ostomy**				**Liver problems (ICD K71–76)**		**Short bowel (ICD K91.2)**		**Operative central venous access**		**Operative feeding tube**	
						**Primary**	**Secondary**	**None**									
	***N* (%)**	***N* (%)**	***P***	**Median (IQR)**	***P***	***N* (%)**	***N* (%)**	***N* (%)**	***P***	***N* (%)**	***P***	***N* (%)**	***P***	***N***	***P***	***N* (%)**	***P***
2007–16	435	22 (5%)		65 (32;115)		260 (59.8%)				71 (16%)		97 (22%)		94 (22%)		51 (12%)	
Male	235 (54%)	16 (7%)	0.081	67 (32;124)	**0.023**	144 (61%)	18 (8%)	73 (31%)	0.699	41 (17%)	0.517	49 (21%)	0.488	39 (17%)	**0.007**	32 (14%)	0.231
Female	200 (46%)	6 (3%)		62 (32;104)		116 (58%)	19 (10%)	65 (33%)		30 (15%)		48 (24%)		55 (28%)		19 (10%)	
Pre-maturity	280 (64%)	19 (7%)	**0.012**	86 (50;137)	** <0.001**	181 (65%)	26 (9%)	73 (26%)	**0.003**	52 (19%)	0.104	65 (23%)	0.631	74 (26%)	** <0.001**	43 (15%)	**0.002**
Cardiac malformations	92 (21%)	8 (9%)	0.194	99 (55;140)	**0.008**	56 (61%)	7 (8%)	29 (32%)	0.983	19 (21%)	0.207	20 (22%)	1	23 (25%)	0.393	18 (20%)	**0.016**
Defects of abdominal wall	69 (16%)	2 (3%)	0.742	81 (46;143)	0.094	39 (57%)	11 (16%)	19 (28%)	0.067	15 (22%)	0.213	30 (44%)	** <0.001**	32 (46%)	** <0.001**	13 (19%)	0.064
Malformations of CNS	35 (8%)	2 (6%)	0.79	93 (41;161)	0.165	24 (69%)	4 (11%)	7 (20%)	0.251	9 (26%)	0.148	8 (23%)	1	8 (23%)	0.832	6 (17%)	0.279
Urogenital malformations	30 (7%)	0	0.438	74 (46; 132)	0.67	25 (83%)	1 (3%)	4 (13%)	**0.026**	6 (20%)	0.608	4 (13%)	0.263	5 (17%)	0.647	5 (17%)	0.377
Cystic fibrosis	29 (7%)	0	0.142	54 (44;76)	**0.019**	21 (72%)	2 (7%)	6 (21%)	0.359	8 (28%)	0.114	5 (17%)	0.646	3 (10%)	0.162	1 (3%)	0.231
Malformations of limbs	24 (6%)	1 (4%)	0.63	86 (48;162)	0.311	18 (75%)	2 (8%)	4 (17%)	0.222	4 (17%)	1	6 (25%)	0.801	10 (42%)	**0.021**	3 (13%)	0.753
Anorectal malformation	8 (2%)	0		71 (24;116)		5 (63%)	1 (13%)	2 (25%)	0.735	0	0.364	2 (25%)	1	3 (38%)	0.378	2 (25%)	0.239
Duodenal atresia	8 (2%)	1 (13%)		49 (22; 129)		3 (38%)	1 (13%)	4 (50%)	0.296	1 (13%)	1	3 (38%)	0.385	3 (38%)	0.378	3 (38%)	0.056
M. Hirschsprung	4 (1%)	0		71 (30;75)		2 (50%)	2 (50%)	0	**0.042**	1 (25%)	0.511	3 (75%)	**0.036**	2 (50%)	0.205	0	1
Biliary atresia	4 (1%)	1 (25%)		51 (38; 91)		2 (50%)	0	2 (50%)	0.728	3 (75%)	**0.015**	1 (25%)	1	1 (25%)	1	0	1
Other chromo-somal defects	4 (1%)	0		91 (71;165)		4 (100%)	0	0	0.512	0	1	2 (50%)	0.216	1 (25%)	1	1 (25%)	0.394
Trisomy 21	2 (1%)	1 (50%)		18		0	0	2 (100%)	0.161	0	1	0		0	1	0	1
Esophageal atresia	1 (0%)	0		64		1 (100%)	0	0	1	0	1	0	1	1 (100%)	0.216	0	1
Necrotizing enterocolitis	42 (10%)	4 (10%)	**0.007**	138 (99;168)	<0.001	31 (74%)	6 (14%)	5 (12%)	**0.005**	10 (24%)	0.186	12 (29%)	0.33	19 (45%)	** <0.001**	7 (17%)	0.311
Volvulus	54 (12%)	3 (6%)	0.84	52 (25;109)	0.524	33 (61%)	3 (6%)	18 (33%)	0.811	9 (17%)	1	13 (24%)	0.729	12 (22%)	0.862	4 (7%)	0.37
Short bowel	97 (22%)	6 (6%)	**0.012**	98 (68: 163)	<0.001	71 (73%)	16 (17%)	10 (10%)	** <0.001**	29 (30%)	** <0.001**	-	-	59 (61%)	** <0.001**	25 (26%)	** <0.001**

*Bold values indicate p < 0.05*.

The initial coding of the JIA in all patients comprised jejunal atresia in 43.0%, ileal atresia in 49.7%, atresia of other part of the small bowel in 10.8%, and atresia of unspecified part in 18.4%. In 80.3% of all patients, a single diagnosis was coded. Two and three diagnoses were coded in 16.7 and 3.0% of patients, respectively.

Defects of the abdominal wall were coded as gastroschisis (Q79.3) in 56 cases, omphalocele (Q79.2) in 11 cases. In two children both codes were provided. A total of 87.0% of children with abdominal wall defects were premature. The distribution of the different atresia types in patients with abdominal wall defects was jejunal in 40.6%, ileum atresia in 56.5%, atresia of other parts of the small bowel in 14.5% and atresia of unspecified parts in 14.5%. Jejunoileal atresia was diagnosed in 55.2% of patients with cystic fibrosis, ileum atresia in 34.5%, atresia of other parts of the small bowel in 13.8%, and atresia of unspecified parts in 24.1% (Table 2). Forty-eight percent of the children with cystic fibrosis were premature. Necrotizing enterocolitis was coded in 9.7% of all patients. Only two children with NEC (4.8%) were not premature (Figure 1).

**Table 2:** Distribution of types of JIA in all patients and different groups of patients.

	**All patients (*n* = 435)**	**Without NEC (*n* = 393)**	**With NEC (*n* = 42)**	**Cystic fibrosis (*n* = 29)**	**Abdominal wall defect (*n* = 69)**
Jejunal atresia	43.0%	44.8%	26.2%	55.2%	40.6%
Ileal atresia	49.7%	49.1%	54.8%	34.5%	56.5%
Atresia of other part of small bowel	10.8%	11.5%	4.8%	13.8%	14.5%
Atresia of unspecified part of small bowel	18.4%	18.1%	21.4%	24.1%	14.5%

**Figure 3:**
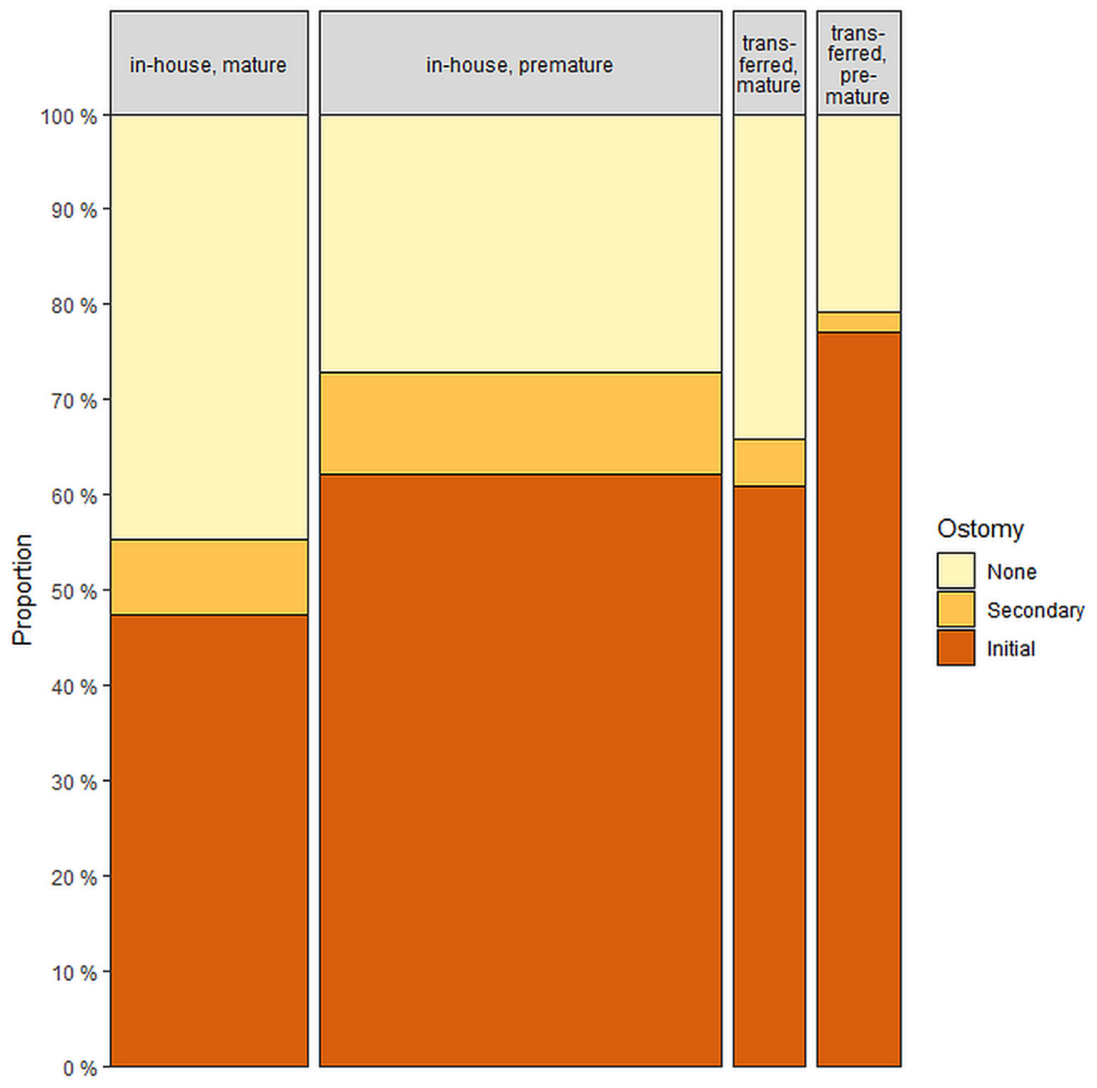
Distribution of patients regarding ostomy with (*n* = 89) and without (*n* = 346) transfer prior to first surgical treatment.

Eighty-nine patients had to be transferred prior to surgery, 48 (53.9%) of whom were pre-mature, three (3.4%) had cystic fibrosis, and two (2.2%) had abdominal wall defect. The median length of stay (LOS) before transfer was 2 days (IQR 1–4). A total of 4.5% were transferred on the day of admission, 22.5% 1 day, 30.3% 2 days, 11.2% 3 days and 31.5% >3 days after the first admission. The median complete LOS was 50 (IQR 30–97) days for all transferred patients. A total of 69.7% of these patients required initial ostomy (Figure 3), and 22.5% developed short bowel. The 1-year mortality post initial surgery was 3.4%, three patients died before discharge.

**Figure 4:**
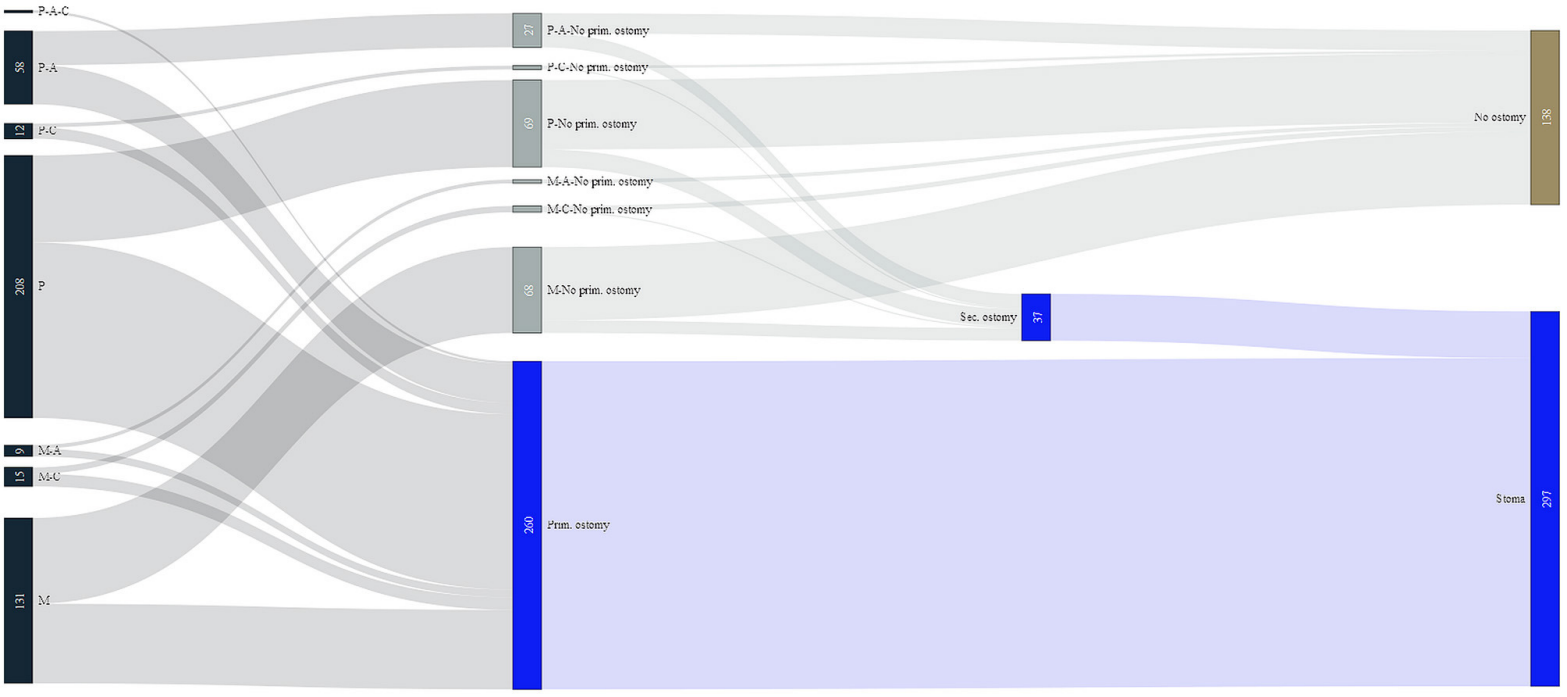
Flow chart for surgery regarding ostomy. P, pre-mature; M, mature; C, cystic fibrosis; A, abdominal wall defect.

A total of 260 patients underwent initial ostomy (59.8%) at the time of first small bowel surgery, 30 of whom underwent ostomy by the Bishop-Koop procedure. Thirty-seven additional patients required ostomy during secondary surgery (Figure 4). In these patients, first surgery consisted of resection of small bowel (16), resection of colon (5), anastomosis small bowel with colon (6), detorsion volvulus (2), and operation of the abdominal wall (9). The median time between first surgery and secondary ostomy was 18.5 days (IQR 8.25–55.75). A total of 31.7% of all patients did not undergo ostomy formation. Of those patients who had diagnosis of NEC during or before first stay only 12% did not undergo ostomy formation. Those patients without any diagnosis of NEC received initial ostomy in 58.3% and secondary ostomy in 7.9%. Incidental appendectomy was performed in 52 cases (12.0%).

Eight patients had initial non-intestinal surgery, and all were related to abdominal wall defect closure. Sixty four patients with abdominal wall defect had complete information regarding time of closure of the abdomen and date of intestinal surgery. Of those 64 patients 54 (84.4%) had intestinal surgery at the date of primary operation of the abdominal wall. 20 (31.3%) had primary anastomosis out of which five needed secondary ostomy, 34 (53.2%) had primary ostomy out of which four were performed as Bishop-Coop ostomy.

**Figure 5:**
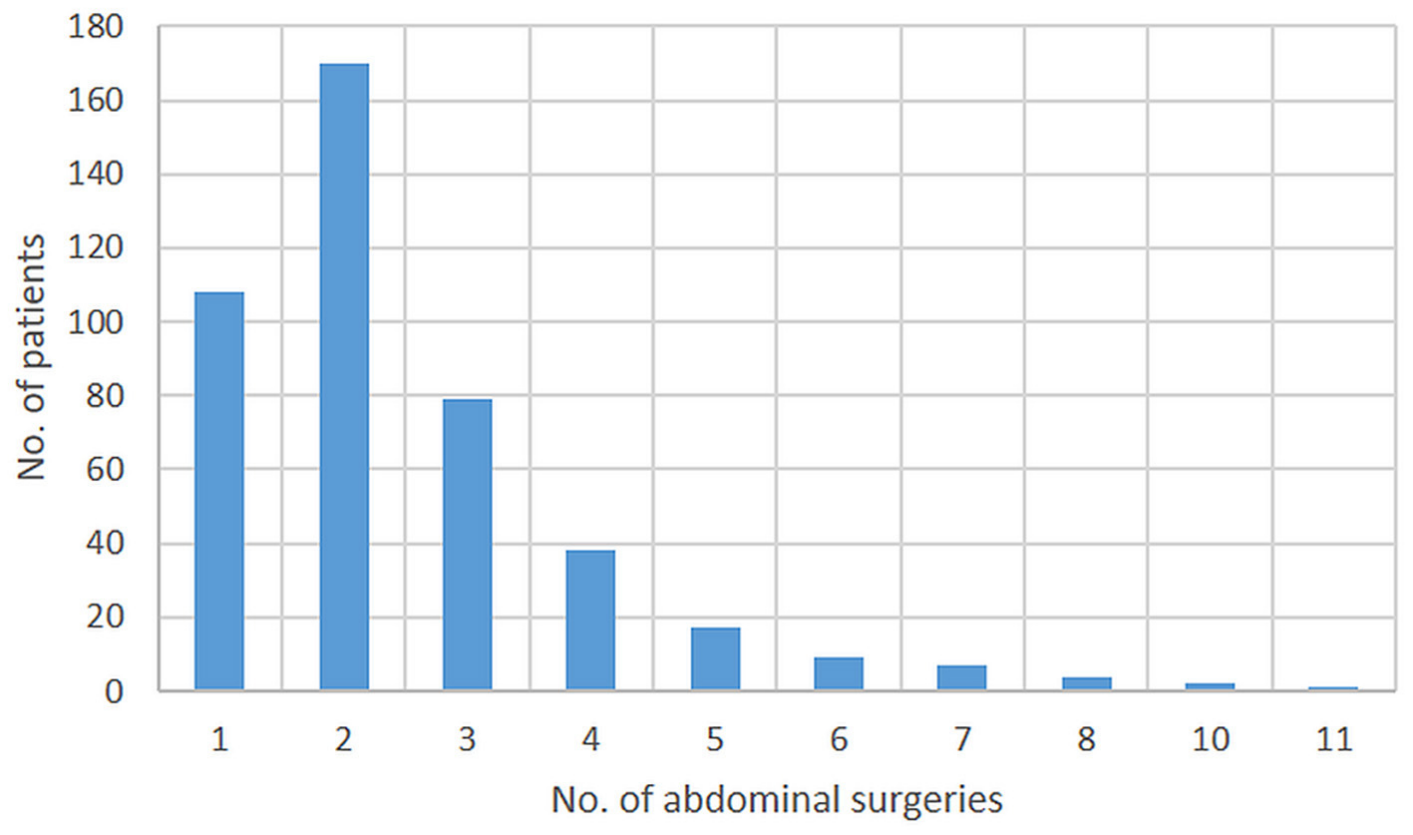
Number of abdominal surgeries per patient.

Follow-up was at least 1 year or until death in all but eight patients. Median follow-up was 4.6 years (IQR 2.0–7.3). During this time, 1–11 surgical intestinal procedures were performed during the follow-up period. The distribution is provided in Figure 5.

In 22.3% of the patients, malabsorption after surgery was documented, which is the same code as that for short bowel (ICD K91.2). 25.8% of these children required a feeding tube, and 60.8% of them required a permanent central venous line (Table 1). Six children (1.4%) required the STEPS procedure, two of them twice and one three times, and another two children required longitudinal lengthening (Bianchi procedure).

In 16.3% of all patients and 29.9% of the patients with reported short bowel syndrome, chronic liver failure, cholestasis, and hepatorenal syndrome (ICD K71-K76) were documented. One liver transplantation but no small-bowel transplantation was performed in this patient group. The patient with liver transplantation also had biliary atresia but no short bowel syndrome.

The median LOS of the first stay for all patients was 66 days (IQR 32–115). Significantly longer LOS was found in boys, premature children, patients with associated cardiac malformations, and those who developed short bowel or necrotizing enterocolitis. A significantly shorter LOS was observed in children with cystic fibrosis (Table 1). The median LOS was 81 days (IQR 48–126) for patients with primary ostomy, 86 days (IQR 59–159) for patients with secondary ostomy and 32 days (IQR 22–61) for patients without ostomy. This difference was significant (*p* = 0.003).

One year after initial surgery, the overall mortality was 5.1%. Specifically, the overall mortality rates were 6.8% in boys and 3.0% in girls, but this difference was not significant. Higher mortality was related to pre-maturity, necrotizing enterocolitis and short bowel. A total of 14/29 patients died during the first stay, and 15/29 died within 1 year after the initial surgery. Of those two who died late, one had short bowel syndrome and renal failure, and the other died of a surgical problem.

The comparison of the three groups with and without ostomy is displayed in Table 3.

**Table 3:** Comparison between patients with and without ostomy.

	**Total**		**Ostomy (Initial)**		**Ostomy (secondary)**		**No Ostomy**		***p***
	**435**		**260**	**59.8%**	**37**	**8.5%**	**138**	**31.7%**	
Necrotizing enterocolitis	42	9.7%	31	11.9%	6	16.2%	5	3.6%	**0.005**
Volvulus	54	12.4%	33	12.7%	3	8.1%	18	13.0%	0.811
Short bowel (ICD K91.2)	97	22.3%	71	27.3%	16	43.2%	10	7.2%	** <0.001**
Liver problems (ICD K71–76)	71	16.3%	46	17.7%	8	21.6%	17	12.3%	0.237
Operative central venous access	94	21.6%	61	23.5%	17	45.9%	16	11.6%	** <0.001**
Operative feeding tube	51	11.7%	24	9.2%	11	29.7%	16	11.6%	**0.004**
Mortality 1 year after initial surgery	22	5.1%	13	5.0%	1	2.7%	8	5.8%	0.840
LOS (IQR)	66 (32;115)		81 (48; 126)		86 (59; 159)		32 (22; 61)		**0.003**

*Bold values indicate p < 0.05*.

## Discussion

In absence of a national registry large data-sets on congenital malformations are difficult to obtain. We could show in a previous study that insurance data are a reliable source for epidemiologic and outcome studies (4). However, missing clinical data are the main limitation of these studies. With the dataset of the AOK, which is the biggest health insurance company in Germany, covering about a third of the German population, we could present the largest cohort of JIA in a single study in Germany. This is the main strength of this study.

This retrospective observational study shows the epidemiology and outcome parameters of 435 children with JIA over a 10-year period. In contrast to other studies (17, 18), concomitant diseases, such as abdominal wall defects or volvulus, were deliberately included in the analysis. The use of administrative claims data includes the risk of a coding error. In Germany, coding rules allow the use of suspected diagnoses, which does not require a formal confirmation later. We avoided the inclusion of these false coded patients by always using the data combination of the ICD-Code of small bowel atresia together with an OPS-code for surgery of ileum or jejunum. Nevertheless, if a child has multiple diagnoses related to the intestinal tract, these procedures can be related to diagnoses other than JIA. This risk was seen in the group of patients with NEC. Administrative claims data cannot reliably differentiate between those patients who develop NEC after operation of JIA or patients where the code of JIA is used for a stenosis after development of NEC. Therefore, further analyze of the different subgroups of patients (NEC, abdominal wall defects, cystic fibrosis) was provided.

The incidence of JIA of 2.1 per 10,000 live births and 1.9 per 10,000 live births in the group without NEC is higher than that in other reports with rates between 0.54 and 1.6 (1, 18–20). Persons insured at AOK have lower socio-economic status compared to persons insured elsewhere (10). Socio-economic status of the mother can have an effect on the incidence. In a study from US mothers of cases with birth defects were more likely to be obese, have Type 1 or 2 diabetes mellitus and smoke tobacco (21). The higher incidence could be related to the abovementioned inclusion criteria of including concomitant diseases, which were excluded in other studies.

The male:female ratio of 1.2 is in the range of other studies (18, 22, 23). The rate of premature children (64.4% in all patients, and in 61.1% in the group without NEC) was greater than that in other studies, where it was reported to be between 34 and 55% (17, 20, 24, 25). This finding could be related to the abovementioned inclusion criteria given that patients with abdominal wall defects tend to be born prematurely. Another reason can be seen in the fact that some studies provide study populations which start in the 70th of last century (17, 24) where the rate of pre-mature children treated was lower than today. One study from South Africa that focused on apple peel atresia reported an even higher rate of premature children (82.4%) (26).

A total of 20.5% of the patients in our cohort had to be transferred before the operation, and 53.9% of them were premature. In Germany, legal regulations divide perinatal centers into three groups. Legal regulations require pediatric surgery at high care perinatal centers. Premature children or those with prenatally detected congenital malformations should be transferred before birth and delivered in a high care perinatal center. Given that our data do not provide the affiliations of the hospitals, the reason for the transfer (no pediatric surgeon at the hospital or delivery outside a perinatal center) remains unclear.

Surgery for JIA in Germany reveals an obvious concept of performing primary ostomy in most patients (59.8%). This finding is clearly different from the literature where the rate is between 11 and 42% (17, 25, 27–29). One reason for the higher rate of stoma formation could be seen in the number of patients with NEC who determine 10% of the analyzed patients. But the analyze of patients without NEC reveals a rate of primary ostomy of 58.3%, which is nearly the same as for the whole group.

In our group, patients who needed a stoma significantly more often needed operative central venous access and an operative feeding tube. Short bowel was coded more often in these children, and they had a significantly longer LOS. This indicates that the group of patients without stoma is the group with less severe medical problems. These findings have to be analyzed further in a clinical study, especially to determine subgroups with higher risks or to define recommendations which patients should be treated with stoma or primary anastomosis. In contrast to the clinical paramters the group of patients who needed a stoma revealed lower mortality than those without, but this finding was not significant. There are sparse data on this topic in the literature, but the publication with lowest rate of initial stoma at only 11.6% also has a low mortality (27).

Previous studies have shown an increased prevalence of cystic fibrosis in JIA patients of up to 12% (24, 25, 28–30). In our data, the rate of cystic fibrosis was 6.7%. Interestingly, these children had a significantly shorter LOS (54 instead of 66 days). No mortality was noted in this group. It could be hypothesized that intestinal problems are not leading issues in patients with CF.

A total of 15.9% of the patients had abdominal wall defects, which is consistent with the current literature (2, 29). Surgery of the abdominal wall defect can be performed prior to the repair of atresia (2). This was performed in 11.6% of our patients. In most patients surgery of the abdominal wall was accompanied with surgery for the atresia. Patients with abdominal wall defects developed short bowel approximately twice as often as the entire remaining group. Only two children died in this group, but the lower mortality rate was not significant.

The LOS of our patients was greater than that in other studies. International studies show a median LOS between 16 and 32 days (16, 17, 25) or an average LOS of 41 days (2). In contrast, we had a median LOS of 66 days. One reason for this finding is that we counted the entire LOS, including transfers to pediatric wards, while other studies only counted the LOS of the surgical cases (17).

Mortality in our cohort was 5.1%. That is lower than that in many other studies, where it was up to 31% (16), but mostly between 10 and 16% (24, 25, 28, 29). Two additional studies had a mortality rate between 3.4 and 4.7% (27, 31).

In our study, short bowel was coded in 22.3% of patients, which is at the upper end of available data in the literature (25, 29, 32), but only 1.8% of all children required intestinal lengthening. This finding is similar to that reported in the literature (2, 29).

Our study showed differences to literature especially for the high rate of ostomies. The new registry on congenital malformations (33) in Germany will give us the opportunity to perform further research on this aspect.

## Conclusion

The outcome of patients with JIA in our national cohort is good compared to the available literature, especially for mortality. A clear difference is the increased rate of ostomies performed. As our data do not allow to give recommendations for the initial surgical approach, this finding should be analyzed further in a clinical follow-up study.

## Data Availability Statement

The datasets for this study cannot be made publicly available because of data protection and legal reasons. In Germany, the utilization of health insurance data for scientific research is regulated by the Code of Social Law. Researchers have to obtain approval from the health insurance providers as well as their responsible authorities. As this approval is given only for a specific research question for a specific time and for a specific group of researches, data cannot be made publicly available. Requests to access the datasets should be directed to http://wido.de.

## Author Contributions

AS, MH, SG, and UR conceptualized and designed the study. AS and MH carried out statistical analyses. AS drafted the initial manuscript. All authors revised and reviewed the manuscript critically and approved the final manuscript as submitted.

## Conflict of Interest

The authors declare that the research was conducted in the absence of any commercial or financial relationships that could be construed as a potential conflict of interest.
